# Prognostic Significance of the Post-Treatment Neutrophil-to-Lymphocyte Ratio in Pharyngeal Cancers Treated with Concurrent Chemoradiotherapy

**DOI:** 10.3390/cancers15041248

**Published:** 2023-02-16

**Authors:** Ji Min Yun, Man Ki Chung, Chung Hwan Baek, Young Ik Son, Myung Ju Ahn, Dongryul Oh, Ki Won Kim, Yoon Kyoung So

**Affiliations:** 1 Department of Otorhinolaryngology-Head & Neck Surgery, Ilsan Paik Hospital, Inje University College of Medicine, Goyang-Si 10380, Republic of Korea; 2 Department of Otolaryngology-Head & Neck Surgery, Samsung Medical Center, Sungkyunkwan University School of Medicine, Seoul 06351, Republic of Korea; 3 Divison of Hematology and Medical Oncology, Department of Medicine, Samsung Medical Center, Sungkyunkwan University School of Medicine, Seoul 06351, Republic of Korea; 4 Department of Radiation Oncology, Samsung Medical Center, Sungkyunkwan University School of Medicine, Seoul 06351, Republic of Korea

**Keywords:** neutrophil-to-lymphocyte ratio, platelet-to-lymphocyte ratio, systemic inflammatory markers, prognosis, pharyngeal cancer, concurrent chemoradiation

## Abstract

**Simple Summary:**

Systemic inflammatory markers, specifically the pre-treatment neutrophil-to-lymphocyte ratio (NLR) and platelet-to-lymphocyte ratio (PLR), have been associated with recurrence and survival in various type of cancers. However, there have been few studies on the clinical significance of their post-treatment values. The aim of this study was to examine the changes in NLR and PLR after concurrent chemoradiotherapy (CCRT) and to determine their prognostic significance in head and neck cancers. The results showed that, after CCRT, the NLR and the PLR increased significantly (*p* < 0.001 in each), and a high post-treatment NLR was significantly related to worse overall survival and relapse-free survival (*p* = 0.013 and *p* = 0.026). This study suggests that post-treatment NLR values may be useful in cancer treatment follow-up.

**Abstract:**

Background: Even though the pre-treatment neutrophil-to-lymphocyte ratio (NLR) and platelet-to-lymphocyte ratio (PLR) are well-established prognosticators in various cancers including head and neck cancers, there have been relatively few studies on the clinical significance of the post-treatment values. This study aimed to investigate the changes in NLR and PLR after concurrent chemoradiotherapy (CCRT) and to evaluate their prognostic significance in pharyngeal cancers. Methods: This study was retrospectively conducted on 461 consecutive patients with primary pharyngeal cancer who had received definitive CCRT. Blood test results before and after CCRT were obtained, and the pre- and post-treatment NLR and PLR were calculated. Patient prognosis was evaluated based on overall survival (OS) and relapse-free survival (RFS). Results: After CCRT, the NLR increased from 2.01 (interquartile range (IQR), 1.53–2.62) to 2.69 (IQR, 1.93–3.81), and the PLR increased from 118.84 (IQR, 92.61–151.63) to 193.19 (IQR, 146.28–262.46). Along with high pre-treatment NLR and high pre-treatment PLR, high post-treatment NLR was also significantly associated with worse OS and RFS (*p* = 0.013 and *p* = 0.026). In addition, patients with a high ΔNLR (i.e., the difference between pre- and post-treatment NLRs) had significantly worse OS and RFS (*p* = 0.013 and *p* = 0.026). However, only a high pre-treatment NLR (hazard ratio (HR), 2.19; 95% confidence interval (CI), 1.17–4.08; *p* = 0.014), age (HR, 2.16; 95% CI, 1.14–4.08; *p* = 0.018), and stage IV (HR, 2.11; 95% CI, 1.15–3.89; *p* = 0.017) were independent prognostic factors for OS in the multivariate analysis. Conclusions: In patients with pharyngeal cancers, following CCRT, the NLR and PLR increased significantly from pre-treatment values. Like the pre-treatment NLR and PLR, a high post-treatment NLR and a significant increase in NLR were also associated with poor prognosis. Further prospective studies are required to prove the independent significance of the post-treatment NLR and PLR.

## 1. Introduction

Recently, systemic inflammatory markers have been reported to be associated with recurrence and survival rates for various cancers, including hepatocellular carcinoma, urinary tract cancer, and biliary tract cancer [1,2,3,4]. These markers are also widely accepted as prognostic factors in head and neck cancers [3,5,6]. Various systemic inflammatory markers have been studied, including neutrophils and lymphocytes [7,8,9,10]. Neutrophils play a major role in inflammation within the tumor microenvironment, and their count can be a marker of systemic inflammation caused by the tumor. They also participate in various stages from tumor initiation to progression and can reflect the tumor progression [11,12]. In contrast, lymphocytes are the key component of the anti-tumor immune response, and lymphopenia has been associated with poor survival outcome in many advanced solid tumors [13]. The neutrophil-to-lymphocyte ratio (NLR) may reflect both opposing factors. Among existing markers, the pre-treatment NLR and pre-treatment platelet-to-lymphocyte ratio (PLR) are well-established prognostic factors in various cancers, including head and neck cancers [5,6,7,14,15]. However, relatively few studies have been conducted on the clinical significance of their post-treatment values [16,17,18].

The incidence of pharyngeal cancer has increased worldwide in the past decade, particularly oropharyngeal cancer, which is associated with human papillomavirus (HPV) infection [19]. Given the high radiosensitivity of HPV-associated pharyngeal tumors, radiotherapy (RT), or concurrent chemoradiotherapy (CCRT), has become a major treatment for pharyngeal cancers, especially in advanced cases [20]. The pharynx contains a large amount of lymphoid tissue, including the tonsils [21]. Lymphocytes are sensitive to radiation, and radiotherapy to the pharyngeal area can cause a decrease in circulating lymphocytes [16,22,23]. Chemotherapy may also lead to bone marrow suppression, contributing to lymphopenia [24]. Lymphocytes are a crucial component of the anti-tumor immune response, and lymphopenia can result in poor survival outcomes for tumors [25]. In this regard, RT or CCRT can significantly affect systemic inflammatory markers such as NLR and PLR.

This study aimed to examine the changes in inflammatory markers after CCRT and to evaluate their prognostic significance in pharyngeal cancers.

## 2. Materials and Methods

### 2.1. Patients and Data

This study was conducted on a cohort of primary pharyngeal cancer patients who had received definitive CCRT in a single center from March 1994 to March 2019. Initially, 709 consecutive patients were screened for enrollment. Only patients with complete remission of the disease were included. Those with residual cancer or a follow-up of <3 months after CCRT were excluded. Patients with other diseases that could affect blood test values were excluded. These included those with synchronous or metachronous malignancies, hematologic diseases such as idiopathic thrombocytopenic purpura, human immunodeficiency virus infection, liver cirrhosis, and active tuberculosis. We also excluded patients with missing analysis items, such as missing blood test data, missing medical records on recurrences, or missing data on follow-up status. Finally, 461 patients were included. For the 461 enrolled patients, the mean radiation dose (±standard deviation) was 6842 Gy (±207 Gy). Most patients (*n* = 446) received cisplatin-based chemotherapy (cisplatin alone in 65.0%, 5-fluorouracil + cisplatin in 12.1%, and docetaxel + cisplatin in 10.6%). A small number of patients (*n* = 15) received cetuximab chemotherapy. This study was approved by the Institutional Review Board of Samsung Medical Center (SMC IRB file no. 2020-06-125-001) and was conducted in accordance with the Declaration of Helsinki. The need for informed consent was waived because of the retrospective study design.

Pre-treatment blood test results that were checked within 4 weeks before the start of CCRT were obtained. In the case of post-treatment blood test results, values were collected 2 weeks after the end of RT, 4 weeks after the end of chemotherapy (CT), and within 3 months of the end of all treatments. The white blood cell (WBC) count, neutrophil ratio, lymphocyte ratio, and platelet count were obtained. The NLR and PLR were calculated by dividing the neutrophil count and platelet count by the lymphocyte count, respectively. The average of the post-treatment values within the data collection period was used for analysis. Demographic and clinicopathological information, including age, gender, smoking history, primary tumor site, clinical stage, and pathology, were obtained from the medical records of the patients. The HPV status was not incorporated in the analysis because it was only available for a limited number of patients in the study. Out of the total of 461 patients, only 77 (16.7%) underwent testing for HPV using p16 IHC or HPV ISH. Among those tested, the majority (87.0%) were found to be HPV-positive. The prognosis was evaluated based on overall survival (OS) and relapse-free survival (RFS). OS was defined as the time from diagnosis to the date of death or last follow-up. RFS was calculated from the date of diagnosis to the date of the first recurrence or the date of death from any cause. Patients were considered censored if they were without events at the end of the follow-up period.

### 2.2. Statistical Analysis

Wilcoxon’s signed-rank test was used to evaluate changes in blood test results after CCRT. Time-dependent receiver operating characteristic (ROC) curve analysis and area under the ROC curve (AUC) values were used to evaluate the optimal cutoff values of the NLR and PLR. The chi-squared test and Mann–Whitney U test were used to compare categorical and continuous variables between 2 groups, respectively. OS and RFS were assessed using Kaplan–Meier estimates. The log-rank test was used to assess the equality of the survival function between different groups. A Cox proportional hazards model with 95% confidence intervals (CIs) was used to assess the effect of the NLR and other clinical/demographic factors. The SPSS software for Windows, version 17.0 (IBM Corporation, Armonk, NY, USA) and R 4.1.3 for Windows (R Foundation for Statistical Computing, Vienna, Austria) were used for statistical analyses. All tests were 2-sided, and *p* < 0.05 was considered to be statistically significant.

## 3. Results

**Figure 1:**
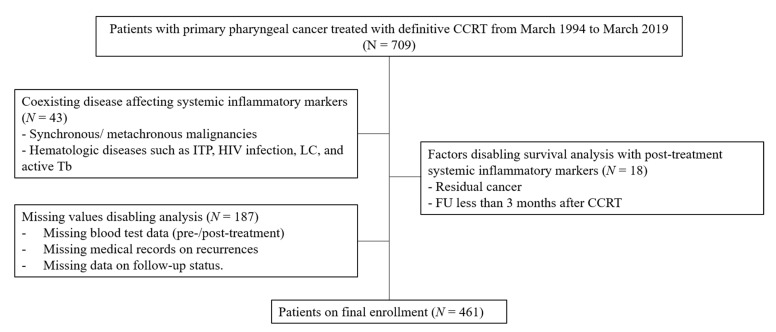
Flowchart of patients’ enrollment.

Among the 709 patients screened on, 461 were finally enrolled in this study (Figure 1).

The clinicopathological profiles of the 461 patients are presented in Table 1. The mean age of the patients was 60.24 years (standard deviation, 13.53 years). There were 380 men (82.4%) and 81 women (17.6%). One hundred and forty patients (30.4%) were smokers. Two hundred and sixty-nine cases (58.4%) had stage IV disease. The median follow-up period was 1102 days (interquartile range (IQR), 444.0–1964.5 days). Among all enrolled cases, 118 (25.6%) had recurrences. The 5-year OS and RFS rates were 86.1% and 70.9%, respectively. The median RFS and OS were 885 days (IQR, 345.5–1809.5 days) and 1163 days (IQR, 514.0–2039.5 days), respectively.

**Figure 2:**
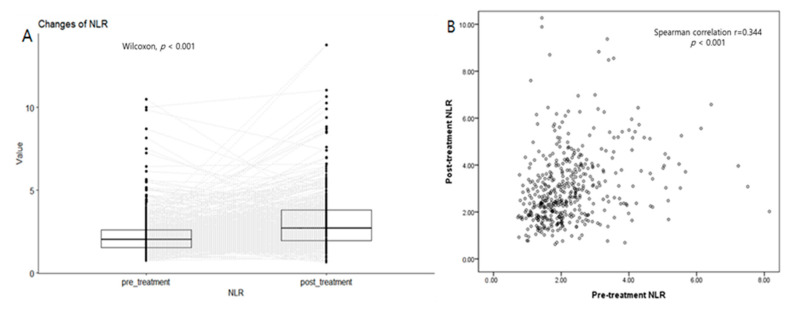
(**A**) Paired box plots of pre- and post-treatment NLR. Each pair of pre-and post-treatment NLR is connected by a dotted line. Post-treatment NLR is significantly higher than pre-treatment NLR. (Wilcoxon singed rank test, *p* < 0.001). (**B**) Scatter plot showed a modest but significant correlation between pre-and post-treatment NLR (Spearman correlation coefficient r = 0.344, *p* < 0.001).

The post-treatment absolute lymphocyte count (ALC), NLR, and PLR were significantly different from their pre-treatment values (*p* < 0.001 in all) (Table S1, Figure 2). After CCRT, ALC decreased from 2039.88 (IQR, 1636.39–2514.86) to 1101.36 (IQR, 865.80–1420.26). In addition, the NLR increased from 2.01 (IQR, 1.53–2.62) to 2.69 (IQR, 1.93–3.81), and the PLR increased from 118.84 (IQR, 92.61–151.63) to 193.19 (IQR, 146.28–262.46). Each post-treatment lab value significantly correlates with the corresponding pre-treatment value (Spearman’s *p* = 0.348 in ALC, 0.344 in NLR, and 0.495 in PLR, respectively; *p* < 0.001 in all).

Based on OS, the optimal cutoffs for pre-treatment and post-treatment NLRs were 2.37 and 3.86 (AUC = 62.4 and 53.7), respectively. Meanwhile, the optimal cutoff values for pre-treatment and post-treatment PLRs were 130.98 and 309.96 (AUC = 62.7 and 52.3), respectively. Patients were divided into two groups based on each optimal cutoff value (Figure S1).

The clinicopathological characteristics according to the post-treatment NLR are shown in Table 2. Patients with high post-treatment NLRs were more likely than those with low post-treatment NLRs to have advanced T classification (*p* = 0.007). Age, sex, smoking history, presence of diabetes mellitus, pathology, N classification, and stage did not differ between the three groups (*p* > 0.05). The baseline NLR and PLR were higher in the high post-treatment NLR group (*p* < 0.001). Similarly, patients with high pre-treatment NLRs tended to have more advanced T classification than those with low pre-treatment NLRs (*p* < 0.001, Table S2).

**Figure 3:**
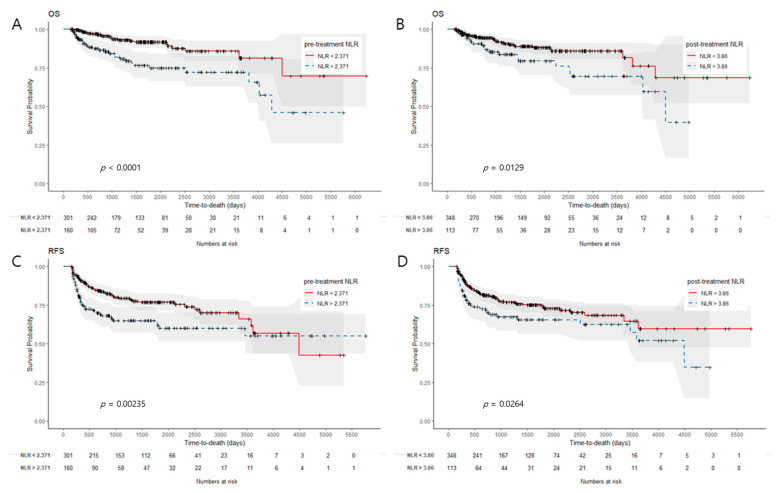
Kaplan–Meier survival curves for OS and RFS according to the different levels of NLR. (**A**,**B**) High pre-treatment and high post-treatment NLR was related to significantly worse OS (*p* < 0.001 and *p* = 0.013, respectively); (**C**,**D**) High pre-treatment NLR and high post-treatment NLR also predicted worse RFS (*p* = 0.002 and *p* = 0.026, respectively).

**Figure 4:**
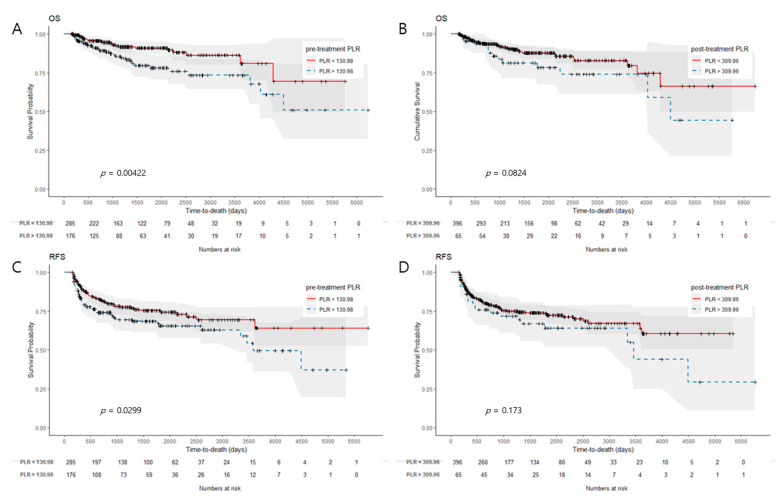
Kaplan–Meier survival curves for OS and RFS according to different levels of PLR. (**A**,**B**) High pre-treatment PLR and high post-treatment PLR was related to significantly worse OS (*p* = 0.004 and *p* = 0.082, respectively); (**C**,**D**) High pre-treatment PLR was related to significantly worse RFS (*p* = 0.030). No significant difference in RFS was observed between high and low post-treatment PLR groups (*p* = 0.173).

Patients with high pre-treatment NLRs had significantly worse OS and RFS than those with low pre-treatment NLRs (*p* < 0.001 and *p* = 0.002, Figure 3A,C). Likewise, those with high post-treatment NLRs had worse OS and RFS than those with low post-treatment NLRs (*p* = 0.013 and *p* = 0.026, Figure 3B,D). A high pre-treatment PLR was also associated with worse OS and RFS (*p* = 0.004 and *p* = 0.030, Figure 4A,C). However, a high post-treatment PLR did not have a significant correlation with worse OS and RFS. (*p* = 0.082 and *p* = 0.173, Figure 4B,D). When patients were stratified based on the difference between pre- and post-treatment NLR (ΔNLR) values (median value, 0.66), patients with high ΔNLRs had significantly worse OS and RFS (*p* = 0.013 and *p* = 0.026). A high ΔPLR was not significantly associated with worse OS or RFS (*p* = 0.082 and *p* = 0.173).

Table 3 demonstrates univariate and multivariate Cox analyses for survival. The univariate analysis revealed that a high pre-treatment NLR, high pre-treatment PLR, high post-treatment NLR, high ΔNLR, patient age, and stage IV disease correlated significantly with worse OS. In the multivariate analysis, a high pre-treatment NLR (hazard ratio (HR), 2.19; 95% CI, 1.17–4.08; *p* = 0.014), age (HR, 2.16; 95% CI, 1.14–4.08; *p* = 0.018), and stage IV disease (HR, 2.11; 95% CI, 1.15–3.89; *p* = 0.017) were independent prognostic factors for worse OS.

In the univariate analysis for RFS, a high pre-treatment NLR, high pre-treatment PLR, high post-treatment NLR, high ΔNLR, and stage IV disease were significant factors. In the multivariate analysis, only stage IV disease (HR, 1.91; 95% CI, 1.28–2.83; *p* = 0.001) and a high pre-treatment NLR (HR, 1.65; 95% CI, 1.08–2.52; *p* = 0.022) were independently associated with worse RFS. 

## 4. Discussion

In this study, ALC, neutrophils, and platelets were significantly decreased from each pre-treatment value after CCRT (*p* < 0.001 each, Wilcoxon signed-rank test). ALC decreased to a greater extent compared to neutrophils and platelets. As a result, post-treatment NLR and PLRs were significantly higher than the pre-treatment values (*p* < 0.001 each, Wilcoxon signed-rank test). This post-treatment change can be affected by the timing of obtaining the lab values after CCRT and chemotherapeutic agents. In this study, we collected the blood lab values of 2 weeks after the end of RT, 4 weeks after the end of chemotherapy (CT), and within 3 months of the end of all treatments. The NLR and PLR were calculated by dividing the neutrophil count and platelet count by the lymphocyte count, respectively, at each time point. Finally, the average of the post-treatment values collected was used for analysis. Chemotherapy-induced neutropenia is generally difficult to predict. The timing and depth of chemotherapy-induced nadirs vary based on patient factors and the type of chemotherapy administered [26]. In this study, most patients received cisplatin-based chemotherapy (cisplatin alone in 65.0%, 5-fluorouracil + cisplatin in 12.1%, and docetaxel + cisplatin in 10.6%). Generally, cisplatin can cause a longer duration of neutropenia than 5-fluorouracil or docetaxel [27]. Cisplatin-induced neutropenia reaches a nadir about 18–23 days after the initiation of chemotherapy. Then, it starts to rise again and may reach a normal level at about 39 days. Thrombocytopenia and lymphopenia can also be caused by chemotherapy. Meanwhile, RT has the greatest effects on the lymphocytes among the erythroid, myeloid, and lymphoid lineages. The lymphocyte LD50 (the lethal dose required to reduce the surviving fraction of lymphocytes by 50%) is 2 Gy [22]. In head and neck cancer, irradiation to the 300–400 lymph nodes and circulating lymphocytes contributes to RT-induced lymphopenia. Compared to chemotherapy-induced neutropenia or thrombocytopenia, this lymphopenia persists for a longer duration. The lymphocyte count begins to decrease during CCRT and recovers slightly around 3 months after treatment [28]. However, a significant number of patients have persistent lymphopenia beyond this period, and some patients develop persistent chronic lymphopenia for years [29,30]. Lin et al. also reported persistent neutropenia, as well as lymphopenia that lasted >12 months after the start of post-operative RT in patients with tonsil cancers [31].

Pre-treatment lymphopenia has been associated with advanced tumor stage and has also been considered a poor prognostic factor [13]. Interestingly, treatment-induced lymphopenia has also been associated with a poor prognosis in cancer patients. In a study involving 403 nasopharyngeal cancer patients treated with CCRT, patients with lymphopenia during CCRT (ALC < 390 cells/mm^3^) and those with lymphopenia 3 months post-CCRT (ALC < 705 cells/mm^3^) had worse 5-year OS and PFS than their counterparts, respectively [32]. A recent meta-analysis including 14 studies showed that severe lymphopenia (ALC < 500 cells/mm^3^) after RT in patients with lung cancer was associated with an increased risk of death (pooled HR, 1.59; 95% CI, 1.40–1.81) and progression (pooled HR, 2.1; 95% CI, 1.57–2.81) compared to patients with no severe lymphopenia [33]. Along with those hematologic changes, NLR and PLR have been generally reported to increase after RT or CCRT. In a study involving 99 patients with tonsil cancers, high acute NLRs (>11.875) and high late NLRs (>6.875) after post-operative RT were independently associated with worse OS (HR, 4.4; 95% CI, 1.2–16 and HR, 12; 95% CI, 3.0–48, respectively) [31]. High post-treatment NLR has been associated with a poor prognosis in lung cancer and breast cancer treated with RT [34,35]. High post-treatment PLR was also reported to correlate with worse PFS (HR, 3.70; 95% CI, 1.07–12.76) and OS (HR, 3.23; 95% CI, 1.01–9.11) in patients with small hepatocellular carcinoma managed with stereotactic body RT [36].

In this study involving 461 patients with head and neck cancers, the patients with high post-treatment NLRs had significantly worse OS and RFS than those with low post-treatment NLRs (*p* = 0.013 and *p* = 0.026). The post-treatment NLR and PLR can have different prognostic significance from the pre-treatment NLR and PLR. They can be used for the follow-up and monitoring of recurrence after treatment, especially given the ease of the tests. Recently, the post-treatment NLR has also been studied as a prognostic factor in the field of cancer immunotherapy. In recent studies involving patients treated with anti-programmed cell death protein 1/programmed death-ligand 1 immunotherapy for non-small-cell lung cancer and small-cell lung cancer, it was reported that patients with high post-treatment NLRs (≥5) had significantly shorter PFS [37,38].

The differences between pre- and post-treatment values also had a significant association with OS and RFS. There have been several reports of the prognostic value of NLR changes. Chen et al. reported that increased NLR (ΔNLR > 0) after two cycles of chemotherapy was associated with a higher risk compared to ΔNLR ≤ 0 (HR, 1.894; *p* = 0.007) in patients with advanced pancreatic cancer managed with chemotherapy [39]. In their study, both the baseline NLR and ΔNLR were independent prognostic predictors. Wang et al. also reported that the post-treatment NLR and ΔNLR were associated with PFS (*p* < 0.001) and OS (*p* < 0.001) in patients with non-small-cell lung cancer treated with RT [34]. The prognostic value of the NLR change in the patients with pharyngeal carcinoma was consistent with previous studies. Along with high pre- and post- treatment NLRs, a high ΔNLR was associated with a poor prognosis.

The pre-treatment baseline NLR and PLR are established prognosticators in various cancers, including head and neck cancers. Consistent with this, this study showed that the pre-treatment NLR and PLR were significantly associated with worse OS and RFS. In the multivariate analysis, a high pre-treatment NLR was an independent prognostic factor for worse OS (HR, 2.19; 95% CI, 1.17–4.08; *p* = 0.014) and worse RFS (HR, 1.65; 95% CI, 1.08–2.52; *p* = 0.022). However, a high post-treatment NLR and PLR were not independently associated with OS in the multivariate analysis. As shown in the results, the post-treatment NLR correlated with the pre-treatment NLR (Spearman’s ρ = 0.344, *p* < 0.001), and the post-treatment PLR with the pre-treatment PLR (Spearman’s ρ = 0.495, *p* < 0.001). This correlation might limit the significance of the post-treatment NLR and PLR in the multivariate analysis. The timing of post-treatment blood tests after CCRT might also have impacted the values of the post-treatment NLR and PLR and their prognostic significance to some extent.

This study has several limitations due to its retrospective design. The timing and frequency of blood tests after treatment varied from patient to patient in this study population. If this study was performed prospectively, it would be possible to obtain the serial values of the NLR and PLR after CCRT on a consistent schedule for all patients. Along with this, the prognostic significance of the labs might be influenced. Another point is that HPV status was not incorporated in the analysis. Because the HPV status is a crucial prognostic factor for pharyngeal carcinomas, incorporating this information into the analysis in a future study would provide more comprehensive results.

## 5. Conclusions

In patients with pharyngeal cancers, following CCRT, the NLR and PLR increased significantly from pre-treatment values. Like the pre-treatment NLR and PLR, a high post-treatment NLR and a significant increase in NLR were also associated with poor prognosis. Further prospective studies are required to prove the independent significance of the post-treatment NLR and PLR.

## Figures and Tables

**Table 1:** Patients’ characteristics.

Variables	Values
**no. of patients**	461
**age**	
**age, *years***	60.24 ± 13.53 *
<60	195 (42.3%)
≥60	266 (57.7%)
**sex**	
male	380 (82.4%)
female	81 (17.6%)
**smoking (%)**	140 (30.4%)
**DM (%)**	50 (10.85%)
T classification **	
1	113 (24.5%)
2	155 (33.6%)
3	120 (26.0%)
4	72 (15.6%)
**N classification**	
0	30 (6.5%)
1	117 (25.4%)
2	289 (62.7%)
3	25 (5.4%)
**stage**	
I	6 (1.3%)
II	52 (11.3%)
III	134 (29.1%)
IV	269 (58.4%)
**follow-up period**, ***days***	1102.0 (444.0–1964.5) ^†^
**OS, days**	1163.0 (514.0–2039.5) ^†^
5-year OS rate	86.1%
**RFS, *days***	885.0 (345.5–1809.5) ^†^
5-year RFS rate	70.9%

OS—overall survival; RFS—relapse-free survival. * Values are expressed as mean ± standard deviation. ** T classification was missing for 1 patient. ^†^ Values are expressed as median (interquartile range).

**Table 2:** Clinicopathological characteristics according to the post-treatment NLR.

Variables	Low Post-Treatment NLR (*n* = 348)	High Post-Treatment NLR (*n* = 113)	*p* Value
**age, *years*** *	61.0 (54.0–69.0)	63.0 (51.0–71.0)	0.815
**male gender**	285 (81.9%)	95 (84.1%)	0.598
**smoking**	109 (32.1%)	31 (28.2%)	0.445
**DM**	37 (10.6%)	13 (11.5%)	0.786
**advanced T (III and IV)**	133 (38.2%)	59 (52.7%)	0.007
**advanced N (II and III)**	234 (67.2%)	80 (70.8%)	0.481
**stage IV (%)**	207(59.5%)	62 (54.9%)	0.387
pre-treatment NLR **	1.91 (1.46–2.43)	2.51 (1.91–3.42)	<0.001
pre-treatment PLR **	111.98 (88.47–142.23)	137.32 (111.32–173.33)	<0.001

Abbreviations: NLR—neutrophil-to-lymphocyte ratio; PLR—platelet-to-lymphocyte ratio. * Values are expressed as mean ± standard deviation. ** Values are expressed as median (interquartile range).

**Table 3:** Univariate and multivariate analysis for survival.

	OS						RFS					
Variables	Univariate Analysis			Multivariate Analysis			Univariate Analysis			Multivariate Analysis		
	HR	95% CI	*p*-Value	HR	95% CI	*p*-Value	HR	95% CI	*p*-Value	HR	95% CI	*p*-Value
**age (≥60)**	2.43	1.30–4.55	0.006	2.16	1.14–4.08	0.018	1.28	0.88–1.86	0.205			
**male gender**	1.36	0.74–3.60	0.228				0.61	0.36–1.06	0.077			
**smoking**	1.05	0.59–1.87	0.858				1.17	0.79–1.73	0.424			
**DM**	1.01	0.40–2.55	0.984				1.06	0.58–1.93	0.853			
**site**												
**stage IV**	2.09	1.15–3.79	0.015	2.11	1.15–3.89	0.017	1.80	1.22–2.66	0.003	1.91	1.28–2.83	0.001
**pre-treatment**												
**high NLR**	2.77	1.62–4.73	<0.001	2.19	1.17–4.08	0.014	1.75	1.21–2.51	0.003	1.65	1.08–2.52	0.022
**high PLR**	2.15	1.26–3.66	0.005	1.47	0.82–2.64	0.198	1.49	1.04–2.14	0.031	1.20	0.80–1.80	0.376
**post-treatment**												
**high NLR**	1.97	1.14–3.41	0.044	1.72	0.83–3.56	0.143	1.55	1.05–2.28	0.028	1.28	0.76–2.16	0.359
**high PLR**	1.68	0.91–3.10	0.096				1.34	0.85–2.12	0.204			
**high ΔNLR**	1.97	1.14–3.41	0.015	0.96	0.81–1.14	0.636 *	1.55	1.05–2.23	0.028	1.03	0.91–1.17	0.614 *
**high ΔPLR**	1.71	0.93–3.15	0.086				1.37	0.87–2.17	0.174			

NLR—neutrophil-to-lymphocyte ratio; PLR—platelet-to-lymphocyte ratio; ΔNLR—difference between pre- and post-treatment NLRs; ΔPLR—difference between pre- and post-treatment PLRs. * ΔNLR and ΔPLR were incorporated into the multivariate analysis as continuous variables.

## Data Availability

The data presented in this study are available on request from the corresponding author. The data are not publicly available due to the institutional policy.

## Supplementary Materials

The following supporting information can be downloaded at: https://www.mdpi.com/article/10.3390/cancers15041248/s1, Figure S1. Receiver-operating curves for ideal cut-off values of pre-/post treatment NLR and PLR; Table S1. Changes in blood lab values after chemoradiation; Table S2. Clinicopathological characteristics according to the pre-treatment NLR.
