# Supplementary material for: Prognostic Significance of the Post-Treatment Neutrophil-to-Lymphocyte Ratio in Pharyngeal Cancers Treated with Concurrent Chemoradiotherapy
Source: Cancers (Basel). 2023 Feb 16;15(4):1248. doi: 10.3390/cancers15041248 (PMC9954210; doi:10.3390/cancers15041248)
Supplement: Supplementary file 1 [file cancers-15-01248-s001.zip › cancers-2204343-supplementary.pdf]

Figure S1. Receiver-operating curves for ideal cut-off values of pre-/post treatment NLR and PLR

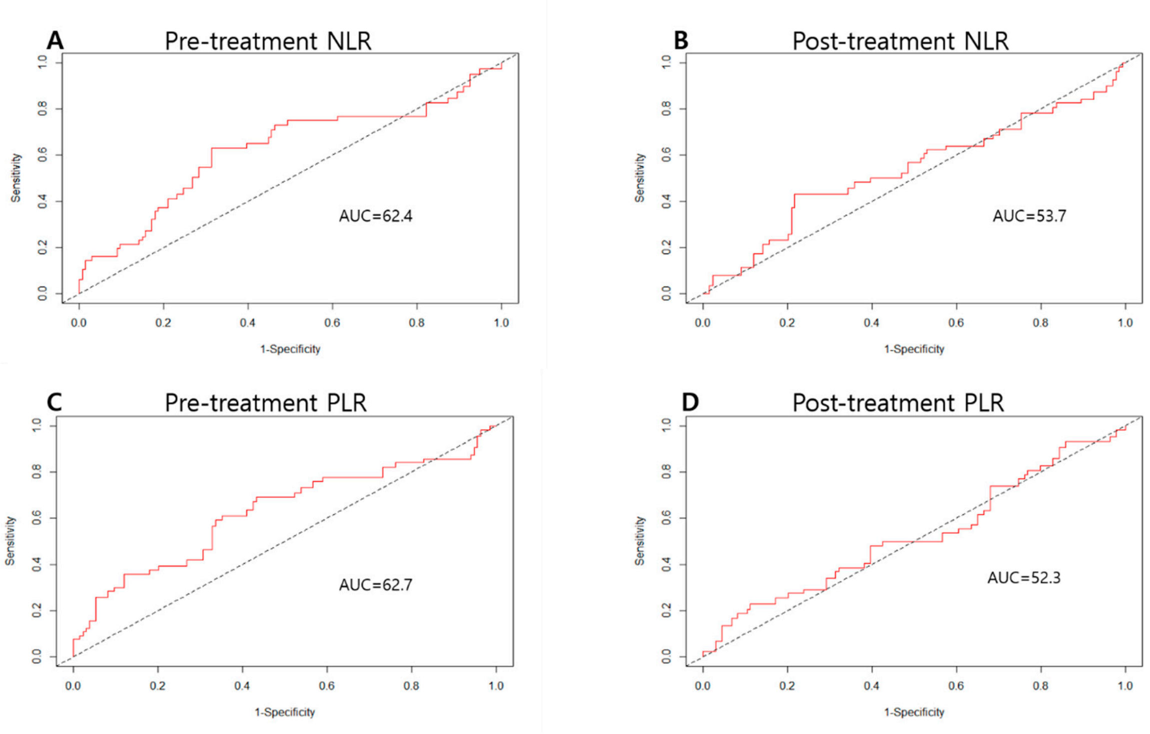

Based on OS, ROC curves for pre-treatment and post-treatment NLRs were drawn (**A&B**, AUC = 62.4 and 53.7). ROC curves for pre-treatment and post-treatment PLRs were also drawn (**C&D**, AUC = 62.7 and 52.3).

Table S1. Changes of blood lab values after chemoradiation

Variables	Pre-treatment	Post-treatment	*p*-Value
**Neutrophil**	4053.80 (3242.9–5204.85)	3001.38 (2300.72–3900.47)	<0.001
**Lymphocyte**	2039.88 (1636.39–2514.86)	1101.36 (865.80–1420.26)	<0.001
**Platelet**	240 (201.00–284.50)	211 (179.00–254.50)	<0.001
**NLR**	2.01 (1.53 –2.62)	2.69 (1.93–3.81)	<0.001
**PLR**	118.84 (92.61–151.63)	193.19 (146.28–262.46)	<0.001

NLR = neutrophil-to-lymphocyte ratio, PLR = platelet-to-lymphocyte ratio.

Values are expressed as median (interquartile range).

Table S2. Clinicopathological characteristics according to the pre-treatment NLR

Variables	Low pre-treatment NLR (*n* = 301)	High pre-treatment NLR (*n* = 160)	*p*-Value
**Age, years***	60.0 ± 13.2	60.7 ± 14.2	0.621
**Male gender**	247 (82.1%)	133 (83.1%)	0.775
**Smoking**	187 (63.2%)	98 (63.6%)	0.923
**DM**	31 (10.5%)	19 (12.3%)	0.550
**Advanced T (III & IV)**	105 (34.9%)	87 (54.4%)	<0.001
**Advanced N (II & III)**	206 (68.4%)	108 (67.5%)	0.837
**Stage IV (%)**	173(57.5%)	96 (60.0%)	0.601
**Pre-treatment PLR****	105.70 (85.05–129.53)	151.25 (118.90–196.54)	<0.001
**Post-treatment NLR****	2.42 (1.81–3.36)	3.35 (2.43–4.47)	<0.001
**Post-treatment PLR****	181.58 (141.03–240.20)	219.53 (156.58–286.78)	0.001

NLR = neutrophil-to-lymphocyte ratio, PLR = platelet-to-lymphocyte ratio.

*Values are expressed as mean ± standard deviation.

**Values are expressed as median (interquartile range).
